# Airway clearance services (ACSs) in Australia for adults with chronic lung conditions: scoping review of publicly available web-based information

**DOI:** 10.1186/s12913-019-4681-1

**Published:** 2019-11-06

**Authors:** Laura Cooper, Kylie Johnston, Marie Williams

**Affiliations:** 1 Southern Adelaide Local Health Network, Intermediate Care Services, Noarlunga Centre, Adelaide, 5168 South Australia; 2 School of Health Sciences, University of South Australia, Adelaide, 5000 Australia

**Keywords:** Airway clearance services, Physiotherapy, Internet

## Abstract

**Background:**

Consumers frequently access the internet looking for health information. With the growing burden of chronic disease internationally, strategies are focussing on self-management interventions in community and ambulatory settings. The objective of this scoping review was to describe publicly available information on Australian airway clearance services.

**Methods:**

Publicly funded health services network webpages and Google were systematically searched between July and November 2018 using relevant keywords. We identified the number, location and currency of contact information of services; and described the services that were in operation and/or identifiable on the internet. Where specific airway clearance services were not identifiable via searching methods, webpages were navigated for associated physiotherapy services. All identified services were contacted via the listed phone or email to confirm web-based findings.

**Results:**

Searching 131 publicly funded health service pages and 191 keyword hits identified four publicly funded airway clearance services (two of which were in operation when confirmed by direct contact) and six private services, all in metropolitan areas of capital cities. Webpages described who their services were for (9/10 services), how to gain referral (4/10) and types of airway clearance techniques available (5/10). A further 286 public physiotherapy services were identified, of which 24 (8%) included descriptors of service provision for respiratory patients on their webpage. In contrast, on direct telephone enquiry airway clearance intervention of some kind was confirmed as being available at 174/286 (61%) sites and unavailable at 69/286 (24%) sites.

**Conclusions:**

This scoping review demonstrated inconsistencies between airway clearance service information available on the internet and the reported provision of services confirmed by direct phone contact. Services that are available need to make information visible to consumers on the internet and include details such as referral pathways, interventions and current contact details, to support people with airway clearance problems to access appropriate care in the community.

## Background

Chronic respiratory diseases, such as bronchiectasis, chronic obstructive pulmonary disease (COPD) and asthma, are the fourth leading causes of death in the world, accounting for more than 3.5 million deaths each year [1]. In 2010, the global prevalence of COPD was estimated to be 384 million (11.7%) people [2], making COPD responsible for 6% of all deaths worldwide [3]. Accurate information on the prevalence of other conditions such as bronchiectasis is developing. The most recent estimates suggest that 1052 per 100,000 people in the United Kingdom (UK) [4], and 1106 per 100,000 in the United States (US) are diagnosed with bronchiectasis [5]. In recent UK primary care data, 42.5% of people with bronchiectasis had a co-existing diagnosis of asthma and 36.1% a co-existing diagnosis of COPD [4]. Globally, high healthcare costs are associated with the management of chronic respiratory diseases. The severity of the disease, the need for inhaled medications, frequent flare ups, repeat hospitalisations and co-existing respiratory conditions significantly increase costs [6, 7]. The cost of inpatient care alone for people with a flare up of bronchiectasis in the US and Spain has been estimated to be equivalent to approximately 11,531 Australian dollars and 7582 Australian dollars respectively [6, 7]. This is greater than the annual cost incurred for COPD management in the US at equivalent to 5995 Australian dollars per person [8].

In Australia, the prevalence of chronic respiratory diseases continues to rise, currently affecting almost one-third (7 million) of Australians [9]. Bronchiectasis, COPD and asthma are all associated with high rates of potentially preventable hospital admissions in Australia, the rates of which increase with remoteness category and socioeconomic disadvantage [10]. COPD alone costs the Australian community an estimated 98.2 billion Australian dollars annually in financial costs, hospital and healthcare costs, lost productivity, premature death and low employment rates [11].

Chronic, and at times, excessive sputum production is a common feature of a variety of chronic respiratory diseases (asthma, COPD, cystic fibrosis (CF) and bronchiectasis). These chronic conditions are characterised by recurrent episodes of flare ups and infections necessitating extensive management from the hospital and primary healthcare sectors. Symptoms of sputum production and associated cough worsen at the time of a flare up, particularly in bronchiectasis [12] or where bronchiectasis coexists with COPD or asthma [13]. In these cases poorer outcomes and increased mortality are seen [14]. People who have frequent flare ups have a faster decline in their lung function and associated quality of life and exercise capacity. They also have a greater likelihood of hospital admission with a longer length of stay compared to those who experience flare ups infrequently [15–17]. In people with COPD, self-management is significantly associated with reductions in hospitalisation rates and breathlessness scores and improvements in health related quality of life [18].

Personalised airway clearance techniques prescribed by a respiratory physiotherapist are recommended in international guidelines for people with bronchiectasis [19–22] and COPD [23, 24] who experience chronic sputum production and/or retention. Such techniques are safe and effective and are recommended both when the person is stable as well as when they are experiencing an acute flare up [25, 26]. Airway clearance services (ACSs) provide assessment and individually prescribed airway clearance techniques to facilitate the efficient and effective expectoration of excess airway secretions in people who are experiencing sputum clearance issues.

Historically these services have been situated in publicly funded hospital-based physiotherapy inpatient and outpatient departments as an adjunct to existing broader services such as pulmonary rehabilitation (PR). Referrals to PR and respiratory physiotherapists are likely to be made by a medical practitioner [27].

The context in which people use health information and seek health services has changed significantly with the evolution of the internet. In US national surveys conducted in 2011–2014 (*n* = 14,128 people) 45–50% of people reported the internet was the first place they sought medical or health information, compared to 10–15% who first sought such information from a health professional [28]. In addition, people who are unable to access care due to delays, insurance issues, or a service not being available at a suitable time had more than twice the odds of using the internet for health information than those who did not report these access difficulties [29]. As such, the information that is available to consumers on the internet needs to be up to date, correct and capture the essence of the service or information being detailed.

## Purpose of this scoping review

This scoping review aimed to describe publicly available web-based information on Australian ACSs and posed the question “What information is publicly available on Australian websites regarding ACSs for adults with chronic lung conditions?

Using only the publicly available information within websites, the key objectives of this review were to:

1. Identify the number, location and currency of contact information for Australian ACSs.
2. Collate and summarise information for Australian ACSs (remoteness classification, health care context, service model, intervention details, resources and contact details.)
3. Where health services did not explicitly indicate the existence of an ACS, identify the presence and currency of information for physiotherapy services, as possible providers of ACS incorporated within another delivery mode.

## Methods

As this scoping review involved direct contact with health sites to confirm currency and confirmation of information, ethical approval was sought and provided by the University of South Australia Human Research Ethics Committee (Approval ID: 201308).

### Defining the scoping review topic

In this scoping review, airway clearance services (ACSs) were defined as ‘A service provided by an individual or specific unit within a health care organisation which provided assessment and individually prescribed treatment plans for facilitating the expectoration of excess airway secretions in people with chronic suppurative conditions. The treatment plan/intervention could include specific education and/or airway clearance techniques, exercise training and/or equipment’. Techniques delivered in a non-acute setting/situation, either as a specific service or as a component of a service were included. Services were eligible for inclusion if they provided ACSs for adults living with chronic lung disease (asthma, COPD and/or bronchiectasis). In Australia, specialised, centre-based health service models for care of people living with cystic fibrosis exist, so ACSs provided specifically for this population were excluded.

Airway clearance techniques could include but were not limited to; education (regarding diagnosis, symptom recognition and role of airway clearance), exercise (to facilitate secretion removal), active cycles of breathing technique (ACBT), forced expiratory techniques (FET), positive expiratory pressure (PEP) and oscillating PEP therapy, non-invasive ventilation, high frequency oscillation and inhalation therapy [30].

### Search strategy and process

Sources of information were restricted to publicly available websites of Australian publicly funded or private health providers. Health related websites for publicly funded providers were identified using the National Health Reform Public Hospital Funding website which provides a list of current local area health networks (LHNs) and their associated hospital sites for each state and territory (site accessed February 2018 at (https://www.publichospitalfunding.gov.au/). For non-government health providers, publicly available search engines (e.g. Google), were used to search for Australian websites of private health providers using key words (airway clearance service, respiratory physiotherapy, chronic lung disease) combined with individual capital cities. Up to the first five pages returned for each webpage were searched using combined key words (Airway clearance service +respiratory physiotherapy +chronic lung disease +‘city).’ National not-for-profit organisations relevant for management of adults with chronic lung conditions were identified by the researchers and searched/navigated for ACS information. In summary, publicly funded health services were searched via specific LHN websites, private health services were searched using Google; with all sites identified (publicly funded or private) contacted to confirm currency of information.

All sites (publicly funded, private and not-for profit) identified by the search strategy were collated in a database (Microsoft Excel Workbook 2010). The specific website for each individual LHN was searched using a standard process. Firstly, where a search function was present in the site, the term ‘airway clearance’ was used (sites without search functionality were searched for appropriate departments or health services (allied health, outpatient and / or respiratory services).Secondly, where an ACS was not identified, webpages specific to allied health, outpatients, respiratory services were searched for any service that potentially incorporated an ACS. This was indicated by any description of a respiratory physiotherapy service that provided airway clearance assessment and/or techniques. Thirdly, where an ACS was identified or services potentially incorporating an ACS (physiotherapy, pulmonary rehabilitation), specific webpages were accessed, saved and printed in hard copy. Given the size and scope of the search, a single reviewer (LC) undertook the search with a random selection (20% of LHNs) repeated by an independent reviewer to assess consistency (KNJ). There were minor inconsistencies found in two (7.7%) of the 26 LHNs in this random sample regarding whether the webpage had a search engine available. Both researchers (LC, KNJ) consulted and reached consensus agreement. There were no other inconsistencies between researchers in this data extraction sample.

Throughout the remainder of the search, where the single reviewer was uncertain about a site, a second reviewer checked eligibility of the material. Websites searches were undertaken between July and November 2018.

## Documentation of the search process

For each website site searched, the name, http address, and date of review was documented. Contact information (including phone, fax and email) for all services (ACS or relevant department /service) was recorded. A single member of the research team (LC) contacted each service / department initially by phone and if unsuccessful, by email. Where three unsuccessful phone attempts were made, and in the absence of email responses, the site was documented as ‘unable to be contacted’. Where contact was successful, confirmation of contact details, currency of website information and clarification of ACS provision (in any form) within the service was sought. Direct contact checking was completed between July and December 2018.

### Data extraction from websites about ACS

A data extraction template was developed a priori and pilot tested with 26 health websites identified within the search by the research team (LC, KNJ, MTW). Where web-based information about an ACS was identified, the following data were extracted (if available) and grouped into a number of domains including:

- *Model/service*: specific health service (listed on the database under the relevant health district / network), geographic location (as per Australian Standard Geographical Classification – Remoteness Area 2006, Australian Government Department of Health [31]), department providing the ACS, stand-alone or integrated respiratory service (nursing, pulmonary rehabilitation) and setting for these services (hospital or community/primary health setting); format of sessions offered (individual versus group)
- *Intended service recipients:* referral pathway, eligible / accepted respiratory diagnoses,
- *Intervention*: details of the types of airway clearance intervention(s) provided within the ACS,
- *Information*: program details, contact person, patient resources, and brochures (availability of resources in languages other than English).

Data was extracted by a single member of the team (LC) verbatim from print versions of all webpages specific to each health provider site.

### Collating, summarising and reporting the findings

Results of the systematic web-based search of publicly funded, private and not-for-profit organisations were summarised in a flow diagram, identifying number of ACSs and number of physiotherapy services found by search engine and web-site navigation. Where no specific ACS was identified but a physiotherapy department was present, the frequency (%) of webpages containing descriptors for respiratory services was reported.

The process and outcomes of telephone confirmation with each ACS or physiotherapy department was summarised descriptively, with regard to accuracy of information (airway clearance service operation and contact details) compared with the webpage entry.

Service characteristics on the webpages of identified ACSs was summarised descriptively. Efficiency of making direct contact for the purposes of confirming web-page information was reported descriptively (frequency (%) of successful contact after single or multiple attempts).

As this was a descriptive scoping review that aimed to provide an overview of the evidence, no methodological appraisal of bias within information returned from searches was planned or undertaken.

## Results

### Search results: identified airway clearance services (named and potential)

**Fig. 1:**
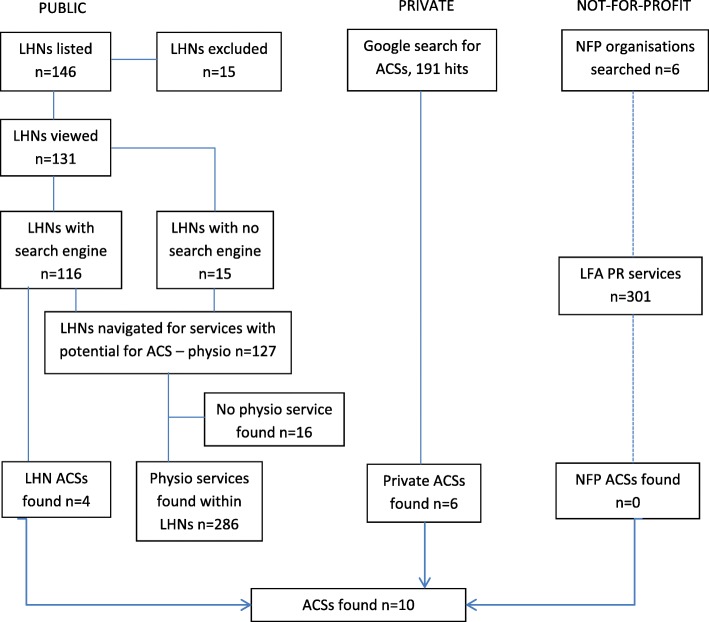
Selection of sources of evidence

Results of the search process to identify ACSs from publicly available websites are shown in Fig. 1. Of the 146 LHNs listed on the 2018 National Reform List, 15 were excluded based on the nature of the service (mental health, forensics or children’s health services, please see Table 1). Of the remaining 131 LHNs websites, 116 included an embedded search function. Of the 116 LHN webpages searched using the term ‘airway clearance’, four webpages directed the viewer to **airway clearance information of some kind** (Table 1).

**Table 1:** Search results: presence of airway clearance services (ACSs) in public, private or not-for-profit health services

	Publicly funded health services (local health network websites)				Private health providers	Not-for-profit organisations
State or Territory	2018 National Reform List *N*=	Excluded *N*=	Included *N*=	ACS^a^ *N*=	ACS identified *N*=	ACS identified *N*=
New South Wales	20	3	17	–	2	–
Victoria	88	7	81	3	–	–
Queensland	19	1	18	–	3	–
Western Australia	7	2	5	–	–	–
South Australia	6	2	4	1	1	–
Tasmania	3	–	3	–	–	–
Australian Capital Territory	1	–	1	–	–	–
Northern Territory	2	–	2	–	–	–
Total:	146	15	131	4	6	–

^a^*ACS* Airway Clearance Service

The Google search strategy for private providers yielded 191 hits from which six private ACSs were identified (Fig. 1, Table 1). Six not-for-profit organisations were searched: Lung Foundation of Australia (LFA), Thoracic Society of Australia and New Zealand, Bronchiectasis Toolbox, the Asthma Foundation, Australian Physiotherapy Association and the Australian Bronchiectasis registry. No airway clearance information or specific services were identified by searching the webpages of these not-for-profit organisations, however there were 301 Pulmonary Rehabilitation programs identified on the LFA website. The combined results from LHN and Google searching resulted in **ten ACS sites.**

Where LHN webpages did not have a search engine, or searching (using the term airway clearance) did not identify airway clearance information, the webpage was navigated to identify physiotherapy services. This method identified 286 publicly funded physiotherapy services across Australia (Fig. 1). A description of services provided for people living with respiratory conditions was found in 24 (8.3%) of these physiotherapy service webpages (Table 2). Common descriptors for respiratory physiotherapy services were; “respiratory outpatients”, “respiratory complications”, “cardiorespiratory”, “breathing problems”, respiratory disease management”, “airway clearance techniques and breathing exercises”, “sputum clearance” and “lung”.

**Table 2:** Search and direct contact results: presence of public physiotherapy services and confirmation of airway clearance service provision

Publicly funded physiotherapy services					
	Information identified from webpages		Information identified by direct telephone contact		
State or Territory	Physiotherapy services *N*=	‘Respiratory physiotherapy’ services *N*=	Physiotherapy services that provide outpatient airway clearance intervention *N*=	Physiotherapy services that do not provide outpatient airway clearance intervention *N*=	Unable to contact
New South Wales	106	2	70	23	13
Victoria	72	5	38	23	11
Queensland	62	6	41	14	7
Western Australia	16	5	11	2	3
South Australia	15	4	7	1	7
Tasmania	13	2	7	5	1
Australian Capital Territory	0	0	0	0	0
Northern Territory	2	0	0	1	1
Total:	286	24	174	69	43

### Airway clearance service information available on webpages

Information extracted from the webpages of identified ACSs is shown in Table 3. Three of the four publicly funded health services identified operated out of physiotherapy departments at tertiary hospitals with the remaining one ACS being in a community based intermediate care setting. Of the six private ACSs identified, three of these practices advertised multiple locations across the metropolitan area while the remaining three operated out of a single site (total number of private practice locations, *n* = 18, Additional file 1: Table S1). All of the ten (100%) ACSs identified had a contact phone number, three (30%) had a fax number, six (60%) had an email contact listed and only one (10%) had a specific contact name on the identified webpage. All ACSs and their associated locations had an Australian Standard Geographical Classification of Remoteness that indicated they were located in major cities of Australia, with 19/22 (86%) classified as inner metropolitan.

**Table 3:** Information extracted from webpages about identified Airway Clearance Services

	Airway Clearance Service identified	Geographic classification	Type of service	Setting	Session format	Referral pathway	Eligible respiratory conditions	Intervention options	Contact name available	Phone	Fax	Email	Contact details correct	Patient resources available	Brochures available
New South Wales	Service 1	Metropolitan	Stand-alone	Private practice	✓	–	–	–	–	✓	✓	–	✓	–	–
	Service 2	Metropolitan	Stand-alone	Private practice	✓	–	✓	✓	–	✓	–	–	✓	–	–
Victoria	Service 3	Metropolitan	Stand-alone	Public hospital	✓	✓	✓	–	–	✓	✓	✓	✓	–	–
	Service 4	Metropolitan	Stand-alone	Public hospital	✓	✓	✓	–	–	✓	–	✓	✓	–	–
	Service 5	Metropolitan	Stand-alone	Public hospital	–	–	✓	–	–	✓	–	✓	✓	–	–
Queensland	Service 6	Metropolitan	Stand-alone	Private practice	✓	–	✓	✓	–	✓	–	–	✓	–	–
	Service 7	Metropolitan	Stand-alone	Private practice	✓	–	✓	✓	–	✓	–	✓	✓	–	–
	Service 8	Metropolitan	Stand-alone	Private practice	✓	–	✓	✓	✓	✓	–	–	✓	–	–
South Australia	Service 9	Metropolitan	Stand-alone	Public hospital	✓	✓	✓	–	–	✓	–	✓	–	–	–
	Service 10	Metropolitan	Stand-alone	Private practice	✓	✓	✓	✓	–	✓	✓	✓	✓	–	–
	10				9 (90%)	4 (40%)	9 (90%)	5 (50%)	1 (10%)	10 (100%)	3 (30%)	6 (60%)	9 (90%)		

A referral pathway for consumers and health professionals was available on the publicly available webpage in four (40%) of the ACSs and nine (90%) described the respiratory conditions that they accepted for airway clearance assessment. Five (50%) of the services described the different types of airway clearance techniques that were available through the service and eight (80%) reported on the format of the airway clearance sessions, with most suggestive of one to one tailored intervention. Common descriptors for tailored intervention were; “the physiotherapist will tailor your treatment to suit your condition”, “programs are customised and developed for all patients” and “tailored to your needs”. None of the ACSs identified provided patient resources or service brochures.

### Airway clearance service information available via direct contact

**Fig. 2:**
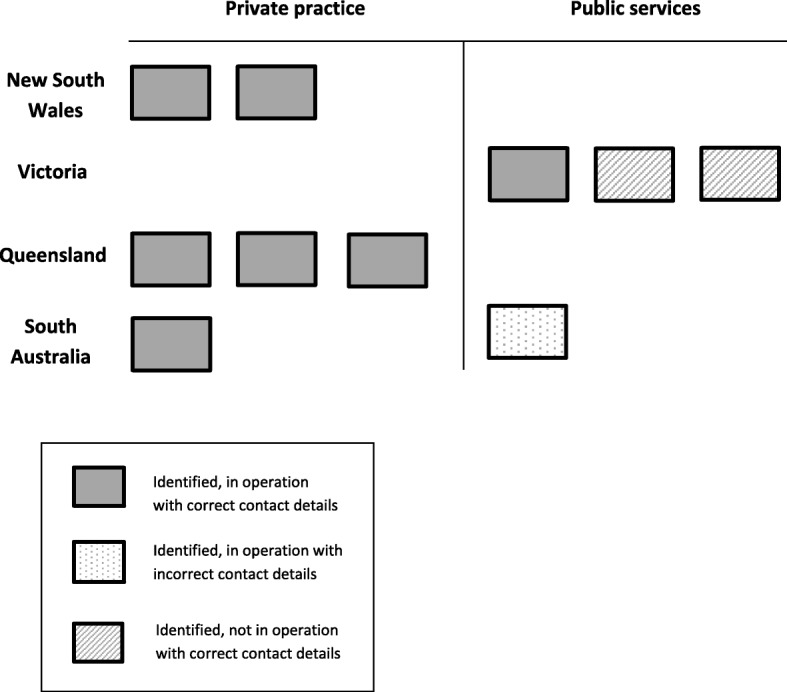
Airway clearance services (ACSs) identified via LHN webpage searching (public service) and google search (private practice) by capital city, currency of service provision and contact information

Direct contact was made to 240 of the 296 identified sites to determine ACS provision and currency of their contact details (LHN searching (*n* = 4), Google searching by capital city (*n* = 6), navigation (*n* = 230)). Fifty-six physiotherapy services (mainly in remote locations with outreach physiotherapy services) were not contacted as the relevant information was gained from contacting another physiotherapy service within the LHN. Of the four ACSs identified via LHN webpage searching, two services in Victorian publicly funded hospitals were currently not in operation due to funding issues (Fig. 2).

Table 2 reports the findings from direct contact with physiotherapy services identified through LHN webpages, to confirm currency of information and presence/absence of ACS provision.

The assessment and prescription of airway clearance techniques either as a stand-alone service or integrated in to a general outpatient physiotherapy service was confirmed as being available at 174/286 (61%) sites and unavailable at 69/286 (24%) sites. Four (16.7%) of the 24 services did not currently provide ACSs for people with respiratory conditions in contrast to information provided on their associated service webpages. Service provision at the remaining 43/286 (15%) sites was unclear due to the researchers being unable to contact the site.

Based on direct contact, via phone and email, it was determined that 294/296 (99%) of the identified sites had the correct contact details displayed on their webpages. In 29/296 (9.8%) of these cases the staff member noted that while the contact information was correct there was a different phone number that would make contact with the airway clearance or outpatient service more direct.

### Accessibility of services by direct contact

Of the 240 ACSs and physiotherapy sites contacted in total, we were able to obtain the desired information within one attempt of contacting the service in 111/240 (47%) cases, within two attempts in 72/240 (31%) cases and within three attempts in 51/240 (22%) cases. A direct phone line to physiotherapy or allied health was present in 30/230 (13%) physiotherapy services identified via webpage navigation.

## Discussion

The prevalence and associated healthcare costs of chronic respiratory diseases is high and is increasing in Australia and internationally due to advances in diagnostic tools, an ageing population and ongoing exposure to identified risk factors [32]. A key finding from this scoping review was that very few designated ACSs were identifiable on the internet and even fewer were in operation. Identified services were located in metropolitan areas and were not available in regional and remote locations where the need for services is greater [33]. There were a great number of physiotherapy services that prescribed airway clearance techniques of some kind but this information was not readily available to the public. While the little information that was available on the internet was mostly correct it did not compare to the scope of service information gained from liaising with physiotherapists.

## Implications for health service delivery

Using conservative estimated prevalence rates for bronchiectasis, a country such as Australia with a population of 25 million may include 263,000 people with bronchiectasis [4]. A conservative approach to current airway clearance recommendations (i.e. four consultations/year/affected person), would require more than one million physiotherapy sessions annually to meet this demand across Australia. This would equate to around three times a full-time equivalent workload, dedicated only to airway clearance, at all of the 184 identified services.

This scenario highlights three major problems. Firstly, the majority of services we contacted operate airway clearance on an ad hoc basis and do not have regular funded clinic facilities to see these people; they are more realistically able to see 1–2 people each week, and unlikely to dedicate three full-time staff to this area every week of the year. Secondly, we have been conservative in this estimation which does not account for extra treatment required to manage flare-ups. Thirdly, as the 184 services identified in this scoping review are not uniformly distributed across Australia there would be areas where service provision and prevalence mal-align.

This review found that the ten ACSs identifiable on Australian webpages were all based in metropolitan areas. This contrasts with high rates of potentially preventable hospitalisations for chronic lung conditions in remote areas such as the Northern Territory (5777 per 100,000 people) in 2016–17 compared to metropolitan Northern Sydney (2032 per 100,00 people) [33]. Remoteness and reduced access to services are key factors contributing to potentially preventable hospital admissions along with affordability, health literacy and socioeconomic disadvantage [10]. The information gained from webpage searching in this scoping review suggests there may be mal-alignment between geographic areas of clinical need and availability of airway clearance services in Australia. This clustering of services in metropolitan areas compared to areas of high disease prevalence outside of capital cities has been described in the chronic heart failure population in Australia, where only 80 (0.7%) of the estimated 16,000 rural chronic heart failure patients were enrolled in disease management programs [34].

## Implications for web-based communication about health services

### This scoping review has highlighted that there is a lack of information available online to consumers regarding ACSs in Australia

There is a significant increase in the number of people accessing the internet for heath related material [35] and in 2015 it was estimated that 78% of Australian adults used the internet to find health-related information [36]. In a sample of 254 Australians with chronic lung diseases who were enrolled in PR programs, 110 (43%) participants used the internet to access health information. Factors associated with increased internet usage for these purposes were age less than 70 years, higher education level and self-reported regular and competent use of laptops and tablets [37]. A further study of technology usage in patients with cardiopulmonary disease in Australia showed that around 15% of the people surveyed accessed disease-specific Facebook support groups for their health information [38]. Results of this scoping review indicate that if consumers rely on the information that is available to them via their LHN webpage, most would assume that an airway clearance service was not provided by their local publicly funded service. In fact, some of these services may be in operation, but the services are simply not described on their webpage. We have demonstrated that in most cases the consumer needs to navigate the LHN webpage to find a physiotherapy service and then make direct contact with the service to ascertain service provision and a referral pathway. Similarly, consulting the webpages of Australian based not-for-profit organisations that focus on the management of chronic lung conditions will provide them with information about availability of pulmonary rehabilitation services but not specifically provide the consumer with where and how to access ACSs.

Making direct phone contact with physiotherapy clinicians in the publicly funded healthcare sector was difficult due to a number of reasons: staff were providing patient care at the time of phone contact; staff were away on leave or the position was unfilled; hospital switchboard staff were often unsure of who was the most appropriate person to contact; and in remote locations the therapists often only visited on certain days of the month in an outreach capacity. Due to these difficulties, the researcher attempted to obtain an email address for any therapist who could answer questions pertaining to airway clearance service provision. Emailing a template of questions enabled a timely, detailed and appropriate response to be received. This method was an acceptable way for the researcher, a physiotherapy clinician, to obtain simple information pertaining to service provision, but may be a potential limitation for consumers due to privacy considerations or lack of familiarity with health service communication systems.

When verbal contact was made with physiotherapy staff in the public funded healthcare sector the researcher found that 20 (11%) of the 174 physiotherapy services prescribing airway clearance techniques were integrating this one-on-one service into their existing PR programs as the need arose. The majority of services operated a daily ad-hoc ACS within a general physiotherapy outpatient department. Service provision for home visiting or community based airway clearance appointments was rare and this information only became apparent through liaising with therapists from larger tertiary sites. The names and contact details of 15 additional private practices, other than the six identified by Google searching, were identified in discussions with publicly funded services that were not available on the internet via our search method. This further demonstrated that not all publicly funded or private ACSs were identifiable on the internet to the consumer despite the service often being available.

## Strengths and limitations of the study

Strengths of this study include conduct by a researcher with clinical experience in respiratory physiotherapy and airway clearance service provision, which facilitated the extraction of relevant information from webpages and when communicating with other clinicians. A potential source of bias in this scoping review was that relevant sources of information may have been inadvertently missed or inaccurately represented. This was minimised by the transparent web-based search process, checked by a second researcher in a random sample, and the added stage of checking in person by direct telephone contact. While two slightly different search methods were employed (i.e. process for searching Local Health Network sites for public providers, and Google search for private providers) each has been transparently described. The telephone contact with every possible identified service or physiotherapy department provided an additional level of confirmation of findings. The scoping review was limited to Australian websites, thus findings may not reflect international practice or health services. Often the discrepancies arose due to different sources of funding, staff allocation and the physical location of services. Study results provide a snapshot of information available and current during the study period (July–December 2018), and specific details may have changed over time.

## Recommendations for future practice and research

An important implication of this scoping review is to ensure that if an airway clearance service is provided, this information needs to be readily available to consumers on the internet. Information should include preferred contact methods and referral pathways. While people with chronic lung conditions were the consumer focus of this scoping review, the findings have implications for making services visible to potential referrers (e.g. general practitioners or physicians) who may use the internet to search for local allied health providers. Further research in this area should consider defining and describing current ACS provision, to assist with national and international benchmarking for models of care.

## Conclusion

In conclusion this scoping review identified a lack of publicly available web-based information about ACSs in Australia. Information about ACSs available online was often inaccurate or incomplete, but this was only identified when direct telephone contact was made with health service providers. Closing the gap between service availability and visibility on the internet may facilitate awareness and access to airway clearance services for people with chronic lung conditions.

## Supplementary information

**Additional file 1: Table S1.** Australian Standard Geographical Classification of Remoteness.

## Data Availability

The de-identified dataset analysed during the current study is available for reproducibility reasons from the corresponding author on reasonable request, subject to and with approval of the overseeing Human Research Ethics Committee.

## Abbreviations

ACBT: Active Cycle of Breathing Technique
ACS: Airway clearance service
CF: Cystic Fibrosis
COPD: Chronic obstructive pulmonary disease
FET: Forced expiratory technique
LFA: Lung Foundation of Australia
LHN: Local health network
PEP: Positive expiratory pressure
PR: Pulmonary Rehabilitation
UK: United Kingdom
US: United States

## References

[1] Naghavi M, Abajobir AA, Abbafati C, Abbas KM, Abd-Allah F, Abera SF (2017). Global, regional, and national age-sex specific mortality for 264 causes of death, 1980–2016: a systematic analysis for the Global Burden of Disease Study 2016. Lancet.

[2] Adeloye D, Chua S, Lee C, Basquill C, Papana A, Theodoratou E (2015). Global and regional estimates of COPD prevalence: systematic review and meta-analysis. J Glob Health.

[3] Global Initiative for Chronic Obstructive Lung Disease (GOLD). Global strategy for the diagnosis, management, and prevention of chronic obstructive pulmonary disease. 2019.

[4] Quint JK, Millett ER, Joshi M, Navaratnam V, Thomas SL, Hurst JR (2016). Changes in the incidence, prevalence and mortality of bronchiectasis in the UK from 2004 to 2013: a population-based cohort study. Eur Respir J.

[5] Seitz AE, Olivier KN, Adjemian J, Holland SM, Prevots DR (2012). Trends in bronchiectasis among medicare beneficiaries in the United States, 2000 to 2007. Chest..

[6] Seitz AE, Olivier KN, Steiner CA, Montes de Oca R, Holland SM, Prevots DR (2010). Trends and burden of bronchiectasis-associated hospitalizations in the United States, 1993-2006. Chest.

[7] de la Rosa D, Martínez-Garcia M-A, Olveira C, Girón R, Máiz L, Prados C (2016). Annual direct medical costs of bronchiectasis treatment: impact of severity, exacerbations, chronic bronchial colonization and chronic obstructive pulmonary disease coexistence. Chron Respir Dis.

[8] Pasquale MK, Sun SX, Song F, Hartnett HJ, Stemkowski SA (2012). Impact of exacerbations on health care cost and resource utilization in chronic obstructive pulmonary disease patients with chronic bronchitis from a predominantly Medicare population. Int J Chron Obstruct Pulmon Dis.

[9] Australian Bureau of Statistics. National Health Survey: First Results. Australia 2014–15 Canberra: ABS; 2015 [4364.0.55.001]. Available from: http://www.abs.gov.au/ausstats/abs@.nsf/mf/4364.0.55.001.

[10] Falster M, Jorm L. A guide to the potentially preventable hospitalisations indicator in Australia. Sydney, NSW; 2017.

[11] Harper E (2013). Lung foundation Australia: promoting lung health and supporting those with lung disease. J Thorac Dis.

[12] Brill SE, Patel AR, Singh R, Mackay AJ, Brown JS, Hurst JR (2015). Lung function, symptoms and inflammation during exacerbations of non-cystic fibrosis bronchiectasis: a prospective observational cohort study. Respir Res.

[13] Quint Jennifer K., Smith Maeve P. (2019). Paediatric and adult bronchiectasis: Diagnosis, disease burden and prognosis. Respirology.

[14] Khoo JK, Venning V, Wong C, Jayaram L (2016). Bronchiectasis in the last five years: new developments. J Clin Med.

[15] Chalmers JD, Goeminne P, Aliberti S, McDonnell MJ, Lonni S, Davidson J (2014). The bronchiectasis severity index. An international derivation and validation study. Am J Respir Crit Care Med.

[16] Pauwels RA, Rabe KF (2004). Burden and clinical features of chronic obstructive pulmonary disease (COPD). Lancet (London, England).

[17] Donaldson GC, Seemungal TA, Bhowmik A, Wedzicha JA (2002). Relationship between exacerbation frequency and lung function decline in chronic obstructive pulmonary disease. Thorax..

[18] Zwerink M, Brusse-Keizer M, van der Valk PD, Zielhuis GA, Monninkhof EM, van der Palen J (2014). Self management for patients with chronic obstructive pulmonary disease. The Cochrane database of systematic reviews.

[19] Polverino E, Goeminne PC, McDonnell MJ, Aliberti S, Marshall SE, Loebinger MR (2017). European Respiratory Society guidelines for the management of adult bronchiectasis. Eur Respir J.

[20] T Hill A, L Sullivan A, D Chalmers J, De Soyza A, Stuart Elborn J, Andres Floto R (2019). British Thoracic Society guideline for bronchiectasis in adults. Thorax.

[21] Chang AB, Bell SC, Torzillo PJ, King PT, Maguire GP, Byrnes CA (2015). Chronic suppurative lung disease and bronchiectasis in children and adults in Australia and new Zealand Thoracic Society of Australia and new Zealand guidelines. Med J Aust.

[22] Visser SK, Bye P, Morgan L (2018). Management of bronchiectasis in adults. Med J Aust.

[23] Yang IA, Brown JL, George J, Jenkins S, McDonald CF, McDonald V, et al. The COPD-X Plan: Australian and New Zealand Guidelines for the management of Chronic Obstructive Pulmonary Disease 2018, Version 2.56, December 2018. 2018. Contract No.: 2.56.

[24] Strickland SL, Rubin BK, Drescher GS, Haas CF, O'Malley CA, Volsko TA (2013). AARC clinical practice guideline: effectiveness of nonpharmacologic airway clearance therapies in hospitalized patients. Respir Care.

[25] Osadnik CR, McDonald CF, Jones AP, Holland AE (2012). Airway clearance techniques for chronic obstructive pulmonary disease. The Cochrane database of systematic reviews.

[26] Lee AL, Burge AT, Holland AE (2015). Airway clearance techniques for bronchiectasis. The Cochrane database of systematic reviews.

[27] Steiner M, Holzhauer-Barrie J, Lowe D, Searle L, Skipper E, Welham S, et al. Pulmonary Rehabilitation: Time to breathe better. National Chronic Obstructive Pulmonary Disease (COPD) Audit Programme: Resources and organisation of Pulmonary Rehabilitation services in England and Wales 2015. London; 2015.

[28] Jacobs W, Amuta AO, Jeon KC (2017). Health information seeking in the digital age: an analysis of health information seeking behavior among US adults. Cogent Soc Sci.

[29] Amante DJ, Hogan TP, Pagoto SL, English TM, Lapane KL (2015). Access to care and use of the internet to search for health information: results from the US National Health Interview Survey. J Med Internet Res.

[30] Main E, Denehy L, Webber B, Pryor JA, Ammani Prasad S. Cardiorespiratory physiotherapy : adults and paediatrics. 0th ed. Edinburgh, [Scotland]: Elsevier Ltd.; 2016.

[31] Australian Government Department of Health. Australian Standard Geographical Classification - Remoteness Area (ASGC-RA 2006) 2019 [Available from: http://www.doctorconnect.gov.au/internet/otd/publishing.nsf/content/ra-intro.

[32] Australian Institute of Health and Welfare. Older Australia at a glance: AIHW; 2018 [Cat. No. AGE 87]. Available from: https://www.aihw.gov.au/reports/older-people/older-australia-at-a-glance/contents/demographics-of-older-australians/australia-s-changing-age-and-gender-profile.

[33] Australian Institute of Health and Welfare. Potentially preventable hospitalisations in Australia by small geographic areas: AIHW; 2018 [Cat. No. HPF 36]. Available from: https://www.aihw.gov.au/reports/primary-health-care/potentially-preventable-hospitalisations/contents/overview.

[34] Clark RA, Driscoll A, Nottage J, McLennan S, Coombe DM, Bamford EJ (2007). Inequitable provision of optimal services for patients with chronic heart failure: a national geo-mapping study. Med J Aust.

[35] Morahan-Martin JM (2004). How internet users find, evaluate, and use online health information: a cross-cultural review. Cyberpsychology & behavior : the impact of the Internet, multimedia and virtual reality on behavior and society.

[36] Australian Institute of Health and Welfare. Australia’s Health 2018 Canberra: AIHW; 2018 [Australia's health series no. 16. AUS 221]. Available from: https://www.aihw.gov.au/getmedia/7c42913d-295f-4bc9-9c24-4e44eff4a04a/aihw-aus-221.pdf.

[37] Seidman Z, McNamara R, Wootton S, Leung R, Spencer L, Dale M (2017). People attending pulmonary rehabilitation demonstrate a substantial engagement with technology and willingness to use telerehabilitation: a survey. J Phys.

[38] Disler RT, Inglis SC, Newton PJ, Currow DC, Macdonald PS, Glanville AR (2015). Patterns of technology use in patients attending a cardiopulmonary outpatient clinic: A self-report survey. Interact J Med Res.

