# Supplementary material for: Airway clearance services (ACSs) in Australia for adults with chronic lung conditions: scoping review of publicly available web-based information
Source: BMC Health Serv Res. 2019 Nov 6;19:808. doi: 10.1186/s12913-019-4681-1 (PMC6836521; doi:10.1186/s12913-019-4681-1)
Supplement: Supplementary file 1 — Additional file 1: Table S1. Australian Standard Geographical Classification of Remoteness. [file 12913_2019_4681_MOESM1_ESM.docx]

Table S1: Australian Standard Geographical Classification of Remoteness

|  | Airway Clearance Service identified | Branches of service | Remoteness Category RA1 (Major Cities of Australia) | Remoteness Classification – Inner Metropolitan | Remoteness Classification – Outer Metropolitan |
| --- | --- | --- | --- | --- | --- |
| New South Wales | Service 1 | Location 1 | ✓ | ✓ |  |
|  |  | Location 2 | ✓ | ✓ |  |
|  |  | Location 3 | ✓ | ✓ |  |
|  |  | Location 4 | ✓ | ✓ |  |
|  | Service 2 | Location 1 | ✓ | ✓ |  |
| Victoria | Service 3 | Location 1 | ✓ | ✓ |  |
|  | Service 4 | Location 1 | ✓ | ✓ |  |
|  | Service 5 | Location 1 | ✓ |  | ✓ |
| Queensland | Service 6 | Location 1 | ✓ | ✓ |  |
|  |  | Location 2 | ✓ | ✓ |  |
|  |  | Location 3 | ✓ | ✓ |  |
|  |  | Location 4 | ✓ |  | ✓ |
|  |  | Location 5 | ✓ | ✓ |  |
|  |  | Location 6 | ✓ |  | ✓ |
|  |  | Location 7 | ✓ | ✓ |  |
|  |  | Location 8 | ✓ | ✓ |  |
|  | Service 7 | Location 1 | ✓ | ✓ |  |
|  | Service 8 | Location 1 | ✓ | ✓ |  |
| South Australia | Service 9 | Location 1 | ✓ | ✓ |  |
|  | Service 10 | Location 1 | ✓ | ✓ |  |
|  |  | Location 2 | ✓ | ✓ |  |
|  |  | Location 3 | ✓ | ✓ |  |
